# Validation of the Spanish version of the Lawton IADL Scale for its application in elderly people

**DOI:** 10.1186/1477-7525-10-130

**Published:** 2012-10-30

**Authors:** Itziar Vergara, Amaia Bilbao, Miren Orive, Susana Garcia-Gutierrez, Gemma Navarro, José María Quintana

**Affiliations:** 1 Primary Care Research Unit-Gipuzkoa, Osakidetza, Spain-Red de investigación en servicios de salud en enfermedades crónicas (REDISSEC), P. Dr Beguiristain s/n, San Sebastian, Spain; 2 Research Unit, Basurto University Hospital (Osakidetza) - Red de investigación en servicios de salud en enfermedades crónicas (REDISSEC), Bilbao, Bizkaia, Spain; 3 Research Unit, Galdakao-Usansolo Hospital- Red de investigación en servicios de salud en enfermedades crónicas (REDISSEC), Galdakao, Bizkaia, Spain; 4 Research Unit, Galdakao-Usansolo Hospital- Red de investigación en servicios de salud en enfermedades crónicas (REDISSEC), Galdakao, Bizkaia, Spain; 5 Public Health and Preventive Medicine Department, Basque Country University (UPV-EHU), Leioa, Bizkaia, Spain; 6 Clinical Epidemiology Unit, Corporacio Sanitaria Parc Tauli, Barcelona, Spain

**Keywords:** Lawton Scale, IADL assessment, Elderly, Validity, Reliability, Responsiveness, Psychometric properties

## Abstract

**Background:**

The adequate information about the functional capacity of elderly populations is a key for the comprehensive assessment of their health status and autonomy. The Lawton IADL (instrumental activities of daily living) Scale is a very often used scale to assess independent living skills, but has never been validated for its use in Spanish-speaking populations. The purpose of this study was to assess the validity, the reliability, and responsiveness of this widely used scale.

**Methods:**

The validation was based on a prospective cohort of 1,965 patients aged 65 or over who suffered an accidental fall with a hip or wrist fracture as a result. These patients were followed up six months after the production of the fracture. Cronbach’s alpha was used to assess reliability, and exploratory factor analysis (EFA) and confirmatory factor analysis (CFA) for the construct validity. Convergent and discriminant validity were examined by the correlation of the IADL Scale with the Barthel Index, SF-12, WOMAC short form, and QuickDASH questionnaires. Known-groups validity was also studied comparing IADL Scale according to different groups, and responsiveness was assessed by means of effect sizes.

**Results:**

The mean age was 80.04 years (SD 8.04). The Cronbach alpha coefficient was 0.94. In the EFA, factor loadings ranged from 0.67 to 0.90, and CFA confirmed the homogeneity of the construct. Regarding the convergent validity, all correlation coefficients were higher than 0.40. Significant differences were found according to different groups, supporting known-groups validity. Responsiveness parameters showed moderate to large changes (effect sizes, 0.79 and 0.84 among patients classified as worsened).

**Conclusions:**

These results confirm that the Spanish version of the Lawton IADL Scale has excellent reliability and validity and a moderate to large sensitivity to change. This study provides a proper validation, not only of the Spanish version of the Lawton IADL Scale, but also of the original instrument.

## Background

Functional capacity is a complex concept that includes advanced activities, instrumental activities of daily living (IADL), and basic activities of daily living (ADL). These components are hierarchically ordered, an ability to develop activities at the highest levels imply the preservation of the lower levels [1,2]. IADL encompasses a group of activities necessary for living independently and their assessment reflects the degree to which individuals are adapted to their environment. Adequate information about the functional capacity of elderly populations is key for the comprehensive assessment of their health status and autonomy. Quality Instruments oriented to the exploration of functional capacity provide patients and health and social professionals with a powerful tool to know and understand the needs they have to tackle. The Lawton IADL Scale is the most widely used [3] instrument for IADL assessment in elder population. In despite of this, it has never been validated for use in Spanish-speaking populations.

We report a validation, of the Spanish version of the Lawton IADL Scale [4], regarding three key aspects of its performance (validity, reliability, and responsiveness), as a reliable instrument for the measurement of IADL. This work also provides valuable insights regarding the validity of the original scale itself.

## Methods

The validation process was based on data from a prospective cohort study of patients aged 65 years or more who attended the Emergency Room (ER) of seven public teaching hospitals for a hip or wrist fracture due to a fall during the period from 2009-3-01 to 2010-01-31. Osakidetza, the Basque Health Service, provides near-universal public health coverage to 2 million people to the autonomous region in northern Spain. All patients were informed about the study and their interest in taking part of it was recorded; those who took part gave their informed consent before inclusion. The study was approved by the Ethics Committee of all participating hospitals. Patients suffering psychological impairments and those where syncope was identified as cause of the fall were excluded. Patients who completed less than 50% of the questionnaires were considered losses. Patients admitted to the hospital were interviewed during their stay and those sent home were interviewed by telephone as soon as feasible thereafter in order to recall the information as close in time to the accident as possible. The follow-up questionnaires at 6 months were sent to all the participants by mail, and in order to avoid losses, participants were carefully followed-up. Specifically, participants were telephoned to increase the response rate and, as required, to adapt the interview procedure to the preferences of the participants, conducting the questionnaires over the phone for those with visual impairments, for example.

The study was based on a comprehensive assessment of patient well-being, including their functional capacity and other relevant variables. Degree of dependence, using the Lawton IADL Scale and the Barthel Index, both before the fall (retrospectively) and at the moment of the interview, self-reported level of health-related quality of life (HRQoL) (using the SF-12 Health Survey, and specific questionnaires such as the short form of the WOMAC (for patients with hip fractures) and the QuickDASH questionnaire (for those with wrist fractures)) were assessed.

The Lawton IADL Scale [5] is composed of 8 items and assesses a person’s ability to perform tasks such as using a telephone or handling finances. Responses to each of the eight items in the scale are coded as 0 (unable or partially able) or 1 (able), and the responses are summed. The summary score ranges from 0 (low function, dependent) to 8 (high function, independent).

The Barthel Index [6] consists of 10 items that measure a person's activities of daily living. The overall possible score ranges from 0 to 100, with lower scores indicating more severe disability. Regarding the final score, patients’ degree of dependence was classified in four groups: < 20 total; 20–35 severe; 40–55 moderate and ≥60 mild dependence. The SF-12 Health Survey [7] is a generic instrument for measuring HRQoL. It contains 12 items from the SF-36 Health Survey [8], to reproduce the physical component summary (PCS) and the mental component summary (MCS), ranging from 0 to 100, with higher scores indicating better level of health. Its validity has been demonstrated in Spanish patients [9].

The WOMAC short form (WOMAC-SF) (Western Ontario and McMaster Universities Osteoarthritis Index) [10] is a shortened version of the self-administered questionnaire [11,12] based on 11 items, to assess pain and function in patients with hip or knee impairments. This scale domains range from 0 to 100, with higher scores indicating worse health status.

The QuickDASH is a shortened version of the DASH Outcome Measure (DASH stands for Disabilities of the Arm, Shoulder and Hand) (Canadian Institute for Work & Health and the American Academy of Orthopaedic Surgeons [AAOS]). Instead of 30 items, the QuickDASH uses 11 items to measure physical function and symptoms in individuals with any of several musculoskeletal disorders of the upper limb. It’s based on a 5-point Likert scale (1–5). Subsequent raw scores range from 30 to 150 and are converted to a percentage, 0 (no disability) to 100 (most severe disability). The QuickDASH also has two four-item optional modules that are scored separately. The Spanish translation of the questionnaire was used [13].

### Statistical Analysis

The description of the variables was carried out using frequency tables, means and standard deviations (SD).

Regarding reliability, the internal consistency of the Lawton IADL Scale was assessed using Cronbach’s alpha coefficient [14], with a value of >0.70 being considered acceptable [15]. The construct validity was studied by exploratory factor analysis (EFA) to test the hypothesis that the eight items on the questionnaire represent a single factor. Items with factor loadings and communalities ≥0.40 were considered acceptable [16]. Confirmatory factor analysis (CFA) for categorical variables was also performed. Different fit indices were evaluated [17-21]: (a) the root mean squared error of approximation (RMSEA) (<0.08 acceptable); and (b) the Tucker-Lewis Index (TLI) and Comparative Fit Index (CFI) (>0.90 satisfactory).

Historically [22], items regarding food preparation, housekeeping, and laundry are omitted for men, with some justifications [23,24], but without the evaluation of the performance of the scale in this respect. EFA and CFA were also performed separately for men and women to study the stability of the construct validity by sex.

Item convergent and discriminant validity was examined by means of item-scale correlations and by correlation with the other scales (SF-12 PCS score, WOMAC-SF, QuickDASH, and Barthel Index) by means of the Spearman correlation coefficient. Item convergent validity was satisfied if the item-own scale correlation corrected for overlap was ≥0.40, while item discriminant validity was satisfied if an item correlated significantly more strongly with the scale it represented than with other scales. The significance of a difference between correlations was assessed with the standard error of the correlation matrix ( $1/\sqrt{n}$), with the recommended significance criterion of two standard errors [25].

Lawton IADL scale convergent and discriminant validity was examined by correlations with the previously named questionnaires. We hypothesized that the correlation coefficient would be higher than 0.40 with those instruments that measured physical domains (Barthel index, SF-12 PCS score, function WOMAC-SF and QuickDASH), and lower with the SF-12 MCS score. In addition, we established that the strength of correlation between the Lawton IADL Scale and the other measures should be lower than the internal consistency of the Lawton IADL Scale [25]. The Spearman correlation coefficient was used.

Known-groups validity was examined by comparing the Lawton IADL Scale among different groups according to age, Barthel Index, SF-12 PCS domain, WOMAC-SF function subscale and QuickDASH scale. We hypothesized that older patients, those with a lower Barthel Index or SF-12 PCS scores, and those with higher scores on the WOMAC-SF function subscale or QuickDASH scales would have a lower Lawton IADL score. For the comparison among two groups, the t-test was used, and analysis of variance (ANOVA) with Scheffe test for multiple comparisons, when more than 2 groups were available, or the non-parametric Wilcoxon and Kruskal-Wallis tests, respectively, if data did not follow a normal distribution.

Regarding the analysis of the responsiveness of the Lawton IADL Scale, three different evolution pathways were expected: some of them would maintain a level of autonomy-dependence similar to prior to the fracture; in another group the level would deteriorate, not only because of the fracture but also because of their age and comorbidities; and just a few patients would report higher levels of autonomy due to improvements in their environment (better facilities and access to care). Therefore, the responsiveness study was performed separately according to groups defined by the score obtained in the Barthel Index.(<20, 20–35, 40–55, ≥60) as follows: those patients with Barthel Index group at baseline lower than at follow-up were classified as “improved”; those with Barthel Index group at baseline higher than at follow-up were considered “worsened”; and those with equal baseline and follow-up Barthel Index groups were classified as “unchanged”. Means and SDs, as well as ceiling and floor effects were calculated for the Lawton IADL scale at baseline and 6 months after the fall. A paired t-test was used to assess the significance of these changes. Further, to measure the responsiveness, we estimated the standardized effect size (SES), defined as the mean change in score divided by the SD of the baseline scores, and the standardized response mean (SRM), defined as the mean change in score divided by the SD of the change scores [26]. Cohen’s benchmarks were used to classify the magnitude of the effect sizes [27]. We expected a larger SES or SRM in patients classified as “worsened” or “improved” than among those classified as “unchanged”.

All statistical analyses were performed with SAS for Windows statistical software, version 9.1 (SAS Institute, Inc., Cary, NC) and Mplus version 6.1 software (18).

## Results

A total of 2,922 patients were initially assessed for their inclusion in the study. Of these, 2,141 patients fulfilled the inclusion criteria, and 176 of these, did not complete the required follow-up questionnaires. The final number of patients was 1,965 (934 with hip and 1,031 with wrist fractures).

84.53% of the participants were women, and the mean age was 80.04 years (SD 8.04). The mean score on the Lawton IADL Scale was 5.56 (SD 2.84) and on the Barthel Index was 89.37 (SD 19.50). Regarding the degree burden of comorbidity present in the studied cohort, 9.15% of individuals have a Charlson Index value of 0, the most frequent value of this index being 2 (19.23%), and with a 50.74% of patients presenting a value equal or higher than 3.

The internal consistency of the eight items on the questionnaire, assessed by Cronbach’s alpha coefficient, was 0.94. The EFA of the eight items found factor loadings of 0.67 to 0.90 and item communalities of 0.45 to 0.81 (Table 1). The percentage of variance explained by the factor was 70.64%. Regarding the results of the CFA, fit indices were good: the RMSEA was 0.08 and the TLI and CFI were both 0.99. Factor loadings were all statistically significant (p<0.001) ranging from 0.84 to 0.96 (Table 1).

**Table 1:** Results of the Exploratory and Confirmatory Factor Analyses and Item Convergent and Discriminant Validity (n=1965)

**Items**	**Item description**	Exploratory Factor Analysis^*^		**Confirmatory Factor Analysis**^**†**^	**Item-scale correlations**^**‡**^	**Correlations with other scales**^**‖**^
		**Factor loading**	**Communality**	**Factor loading**	***r***	**Range of *r***
Item 1	Ability to use telephone	0.67	0.45	0.84	0.61	0.14 – 0.58
Item 2	Ability to do shopping	0.87	0.75	0.94	0.84	0.16 – 0.69
Item 3	Food preparation capacity	0.89	0.79	0.96	0.82	0.15 – 0.65
Item 4	Housekeeping ability	0.90	0.81	0.95	0.87	0.18 – 0.66
Item 5	Capacity to do laundry	0.87	0.75	0.95	0.82	0.12 – 0.63
Item 6	Independence for transportation	0.77	0.59	0.84	0.75	0.21 – 0.65
Item 7	Ability to manage self-medication	0.75	0.57	0.89	0.69	0.15 – 0.64
Item 8	Ability to handle finances	0.80	0.64	0.90	0.70	0.12 – 0.63

*r*: Spearman correlation coefficient.

^*^ The first factor explained the 70.64% of the total variability.

^†^ Fit indexes: χ^2^ = 282.07; degrees of freedom = 20; RMSEA (90% CI) = 0.08 (0.07 – 0.09); TLI = 0.992; CFI = 0.994.

^‡^ Item total correlation with its own Lawton IADL Scale corrected for overlap.

^‖^ Correlation between Lawton IADL Scale and Barthel Index, SF-12 PCS, WOMAC short form and QuickDASH domains.

Regarding specific analysis performed by sex, EFA and CFA showed similar results. In the EFA of the eight items, factor loadings ranged from 0.64 to 0.86 for men and from 0.68 to 0.91 for women, while item communalities ranged from 0.41 to 0.74 for men and 0.46 to 0.82 for women. The percentage of variance explained by the factor was 62.21% for men and 72.73% for women. The CFA fit indices were again good: the RMSEA was 0.08 for men and 0.06 for women, and the TLI and CFI were both 0.98 for men and 0.99 for women. Factor loadings were all statistically significant (p<0.001) ranging from 0.78 to 0.96 in men and from 0.84 to 0.97 in women.

The item-total correlation coefficients corrected for overlap ranged from 0.61 to 0.87, exceeding the threshold of 0.40 (Table 1), and they were higher than the correlation coefficient of each item with the other domains. On the other hand, the Lawton IADL items correlated significantly more strongly with their own scale than with all other scales in 97.5% of cases. In the remaining 2.5%, the difference between the correlation coefficients with their own scale and with the other scale was at least greater than one standard error.

The correlation coefficients between the Lawton IADL score and scores on the Barthel Index, the SF-12 PCS domain, the WOMAC-SF function subscale, and the QuickDASH were all above 0.40 (range, 0.49 – 0.73), while the strength of the correlation with the SF-12 MCS score was much weaker (r = 0.14). Furthermore, all coefficients were lower than the Cronbach’s alpha of the Lawton IADL Scale.

Regarding the known-groups validity, the differences in the Lawton IADL mean score were statistically significant (p<0.0001) among the different groups according to age, and scores on the Barthel Index, SF-12 PCS domain, WOMAC-SF function subscale, and QuickDASH scale (Table 2). It can be observed that there is an ordered change in the Lawton IADL Scale scores when analyzed by age group, older patients having lower IADL functionality. Also a progressive and directional variation can be observed among groups by Barthel Index and SF-12 PCS scores, with those scoring the lowest on these tests having the lowest Lawton IADL scores. Significant differences with the trend in the opposite direction are observed, when comparing Lawton IADL scores with WOMAC-SF function subscale and QuickDASH scores.

**Table 2:** Known Groups Validity of Lawton IADL Scale According to relevant categories

	**Hip + Wrist**		**Hip**		**Wrist**	
	**(n=1965)**		**(n=934)**		**(n=1031)**	
	**n**	**Mean (SD)**	**n**	**Mean (SD)**	**n**	**Mean (SD)**
**Total**	1903	5.56 (2.84)	903	4.32 (2.95)	1000	6.68 (2.22)
**Age**						
≤75^a^	582	7.18 (1.82)^b,c^	118	6.25 (2.60)^b,c^	464	7.42 (1.48)^b,c^
76-85^b^	823	5.58 (2.77)^a,c^	395	4.64 (2.90)^a,c^	428	6.45 (2.34)^a,c^
>85^c^	498	3.63 (2.75)^a,b^	390	3.41 (2.75)^a,b^	108	4.43 (2.62)^a,b^
p-value		<0.0001		<0.0001		<0.0001
**Barthel Index**						
≤35^a^	65	0.42 (0.56)^c^	52	0.35 (0.48)^c^	13	0.69 (0.75)^b,c^
40-55^b^	99	1.36 (1.61)^c^	80	1.08 (1.21)^c^	19	2.58 (2.39)^a,c^
≥60^c^	1714	6.00 (2.54)^a,b^	758	4.94 (2.72)^a,b^	956	6.84 (2.03)^a,b^
p-value		<0.0001		<0.0001		<0.0001
**SF-12 PCS scale**						
<50	1229	4.71 (2.91)	714	3.86 (2.84)	515	5.89 (2.57)
≥50	601	7.43 (1.41)	149	6.83 (2.01)	452	7.63 (1.09)
p-value		<0.0001		<0.0001		<0.0001
**WOMAC-SF Function Subscale**						
[0–25]^a^	-	-	351	6.44 (2.16)^b,c^	-	-
(25–50]^b^	-	-	261	3.99 (2.59)^a,c^	-	-
(50–100]^c^	-	-	300	2.18 (2.30)^a,b^	-	-
p-value		-		<0.0001		-
**QuickDASH**						
[0–25]^a^	-	-	-	-	855	7.09 (1.79)^b,c^
(25–50]^b^	-	-	-	-	98	4.71 (2.77)^a,c^
(50–100]^c^	-	-	-	-	41	2.88 (2.58)^a,b^
p-value		-		-		<0.0001

^a,b,c^ Superscript letters indicate differences among the respective categories of a variable by Scheffe test for multiple comparison.

PCS: WOMAC physical component summary.

In order to assess responsiveness, “unchanged” and “worsened” patients were considered. “Improved” patients were excluded of this analysis due to the small size of this group (7 individuals). Six months after the fall, the Lawton IADL score had decreased 2.19 points among those patients classified as “worsened” and 0.73 points among those classified as “unchanged”. The percentage of individuals at floor level pre-intervention was 7.06%, and 11.97% had this status at the time of the post-intervention assessment (Table 3). The SES and SRM responsiveness parameters were 0.79 and 0.84 among those classified as “worsened”, indicating a moderate to large change. Otherwise, as expected, the parameters were lower among those classified as “unchanged”, 0.31 and 0.38, respectively, indicating a small change (Table 3).

**Table 3:** Changes and Responsiveness Parameters in Lawton IADL Scale 6 Months after the fall

	**Total**	**Worsened**	**Unchanged**
	**(n=809)**	**(n=113)**	**(n=485)**
**Pre**, Mean (SD)	5.65 (2.87)	3.34 (2.77)	6.58 (2.36)
**Post**, Mean (SD)	4.97 (3.08)	1.15 (1.50)	5.91 (2.60)
**Change**, Mean (SD)	−0.98 (2.12)	−2.19 (2.61)^†^	−0.73 (1.90)^†^
p-value^*^	<0.0001	<0.0001	<0.0001
**% at floor**			
Pre-intervention	7.06	14.41	3.98
Post-intervention	11.97	42.73	4.94
**% at ceiling**			
Pre-intervention	47.37	16.22	61.22
Post-intervention	36.52	0	44.85
SES^**^	0.34	0.79	0.31
SRM^***^	0.46	0.84	0.38

^*^ p-value from a paired t-test to compare whether there were significant differences between pre and post scores.

^†^ Superscripts indicate significant differences between “worsened” and “unchanged” groups by means of the t-test.

^**^SES: standardized effect size.

^***^SRM: standardized response mean.

## Discussion

This large prospective cohort study provides detailed information on the validity, reliability and responsiveness of the Lawton IADL Scale in two different samples of patients (hip fractures and wrist factures), with different expected affection of their IADL capacity.

The analysis included a broad range of aspects of the instrument, from the construct of the questionnaire, for which not only exploratory but also confirmatory factor analysis was used, to its convergent and divergent validity and also its known-groups validity. In all cases, it provided very satisfactory results. The reliability was 0.94 for the eight items, EFA was satisfactory, regarding the CFA, the values for the RMSEA, TLI and CFI were satisfactory and factor loadings were all statistically significant.

The correlation was, as hypothesized, strong, both with those on other instruments that measure activities of the daily living, and on instruments exploring functional capacity. Specifically, the correlation coefficient was higher than 0.40, for the Barthel Index, SF-12 PCS domain, WOMAC-SF function subscale and QuickDASH and lower for the SF-12 MCS domain. As stated in the introduction, functional capacity is a complex and hierarchical construct and this relationship is clinically plausible.

Also, as expected, the test was able to identify differences between groups expected to be different from various perspectives. Known groups validation, comparing the Lawton IADL Scale scores among groups classified by age and scores on other related scales, identified significant differences, with older patients scoring lower on the scale under study. Also low IADL Lawton Scale scores were found in those patients with lower Barthel Index and SF-12 PCS scores. Further, those with higher functional limitation, as assessed through WOMAC or QuickDASH scales, had lower Lawton IADL scores.

This study also provides some responsiveness parameters. The SES and SRM responsiveness results indicate moderate to large changes among those classified as “worsened” and a small change among those classified as “unchanged”. Due to the age of the participants and their previous functional status and level of independence, in many cases the traumatic event studied was followed by deterioration or at most marginal improvement in their IADL capacity. This is a plausible explanation for the poor responsiveness after the surgical or orthopedic procedure received, as assessed by recall using this questionnaire. Nevertheless, the percentage of subjects at the floor level pre-intervention was less than 15%, the threshold for the adequacy of a sample to assess responsiveness [28].

Our study has some limitations. First of all, the study cohort has been selected from an ER environment. This fact may led to some reduction of the generalization of the data. Nevertheless, we found that the selected sample, presents values similar to the Spanish elderly population regarding Barthel Index values, and was slightly older and female than the Spanish mean .The main one is the fact that the evaluation of the IADL status of the patient before the fall was obtained retrospectively as reported by the patient once the fall had already occurred. It is evident that the way that information was retrieved leads to bias in the results. Nevertheless, since a fall is an unexpected acute event it would be extremely complicated to obtain such information in a prospective way. It should also be stated that the translation of the questionnaire this work in based in is the most used and referred one, but, at our knowledge extent the translation and back translation procedure was not reported at the time of its publication. The limitations related to the Lawton IADL Scale itself, also had to be considered. The self-report or surrogate report method of administration rather than a demonstration of the functional task may lead either to over- or under-estimation of abilities. In addition, the instrument may not be sensitive to small, incremental changes in function. Due to the relatively high correlation between the Barthel and Lawton Index some results regarding the responsiveness analysis, may be somehow overrated.

Few studies have been performed to test the psychometric properties of the Lawton IADL Scale [22]. It was originally tested concurrently with the Physical Self-Maintenance Scale (PSMS) [5]. Reliability was established with twelve subjects interviewed by one interviewer with the second rater present but not participating in the interview process. Inter-rater reliability was established at 0.85 [29]. Our study, performed in a much bigger cohort, provides also an interesting reliability index, Cronbach’s alpha coefficient being 0.94.

Another published validation of a translation of the Lawton IADL Scale, performed in a smaller sample, analyzed content and construct validity, reliability, and known-groups validity. The results were consistent with ours, with a Cronbach's alpha of 0.86 and intraclass correlation coefficient of 0.99. The factor analysis showed that one factor was extracted, and the known-groups validity was also supported [30].

The potential gender bias of the Lawton IADL Scale has led to the generation of some non-validated adaptations for its application in men [24]. Regarding this issue, our study provides evidence of the construct validity of the scale, in its whole version, when it is applied to women, but also to men. It would be interesting to assess the adequacy and justification of the accepted adaptation of this scale when applied to men.

## Conclusions

These results confirm that the Spanish version of the Lawton IADL Scale has excellent reliability and validity though its sensitivity to change is moderate.

Our findings support the view that this broadly adopted test, due to its characteristics, both in its original form and also in Spanish, is a reliable instrument for the assessment of IADL in elderly individuals living in the community. Given the spread use of this scale, these validity data will improve the IADL assessment procedure in elderly populations.

## Competing interest

The authors declare to have no conflict of interest regarding the results or conclusions retrieved in this work.

## Authors’ contribution

JMQ, AB, SGG and GN are responsible for the project design and review; MO and SGG are responsible for the data review; AB is responsible for the data analysis; IV, AB and JMQ are responsible for the results analysis and the article writing; all authors have taken active part in the article review and corrections. All authors read and approved the final manuscript.

## Financial support

The institution of one or more of the authors has received grants from the Department of Health of the Regional Government of the Basque Country (2008111016) and the Spanish fund for Health Research (Fondo de Investigación Sanitaria) (co-founder: European Fund for Regional Development, EFRD) (PI08/90294).
